# Re-evaluation of how artemisinins work in light of emerging evidence of *in vitro* resistance

**DOI:** 10.1016/j.molmed.2006.03.005

**Published:** 2006-05

**Authors:** Sanjeev Krishna, Charles J. Woodrow, Henry M. Staines, Richard K. Haynes, Odile Mercereau-Puijalon

**Affiliations:** a Centre for Infection, Division of Cellular and Molecular Medicine, St. George's, University of London SW17 0RE, UK; b Department of Chemistry, Open Laboratory of Chemical Biology, Institute of Molecular Technology for Drug Discovery and Synthesis, The Hong Kong University of Science and Technology, Clear Water Bay, Kowloon, Hong Kong; c Unité d'Immunologie Moléculaire des Parasites, CNRS URA 2581, Institut Pasteur, 75724 Paris Cedex 15, France

## Abstract

There are more than half a billion cases of malaria every year. Combinations of an artemisinin with other antimalarial drugs are now recommended treatments for *Plasmodium falciparum* malaria in most endemic areas. These treatment regimens act rapidly to relieve symptoms and effect cure. There is considerable controversy on how artemisinins work and over emerging indications of resistance to this class of antimalarial drugs. Several individual molecules have been proposed as targets for artemisinins, in addition to the idea that artemisinins might have many targets at the same time. Our suggestion that artemisinins inhibit the parasite-encoded sarco–endoplasmic reticulum Ca^2+^-ATPase (SERCA) PfATP6 has gained support from recent observations that a polymorphism in the gene encoding PfATP6 is associated with *in vitro* resistance to artemether in field isolates of *P. falciparum*.

## The importance of artemisinins as antimalarials

**Figure 1:**
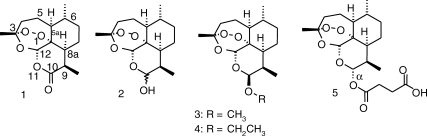
*Qīng hāo su*, also known as artemisinin (1) and derivatives. 2, dihydroartemisinin; 3, artemether; 4, arteether; 5, artesunate. The numbering scheme is that used by Chemical Abstracts (www.cas.org).

Artemisinin derivatives (Figure 1) are uniquely important antimalarial agents. They are useful for treating otherwise resistant parasites [1] and they act faster and over a wider range of parasite developmental stages than other antimalarials [2]. Recently, a study has shown that artesunate reduces mortality compared with quinine in the treatment of severe *Plasmodium falciparum* malaria [3], although this study has raised some interesting issues [4,5]. Like quinine, artemisinin is derived from a plant but is structurally a most distinct compound. The pharmacophore consists of a peroxide within a 1,2,4-trioxane configuration, the presence of which has led to several suggestions of how these antimalarials might work [6]. Many of these suggestions have been based on the understanding of classic peroxide chemistry, and much of the experimental evidence that has been accumulated is based on the chemical behaviour of artemisinins [7,8]. However, the application of medicinal chemistry principles rather than any prior consideration on how the peroxide exerts its antimalarial activity has led to improved forms of artemisinins (both in terms of potency and toxicity) and to the successful synthesis of artemisone [9–12], which is one of the most potent semi-synthetic derivates that is undergoing clinical studies. Experimental chemistry has also generated fully synthetic trioxolanes, which are currently being developed for clinical use [13]. None of these approaches has required knowledge of the mechanisms of action of these drugs.

Understanding how artemisinins work is currently particularly important given the accumulating evidence for artemisinin resistance. The World Health Organization (WHO) has recently underlined this problem by reinforcing previous recommendations that artemisinins should only be used in combination with other antimalarials. Moreover, the WHO publicly requested pharmaceutical companies to end the marketing and sale of ‘single-drug’ artemisinin malaria medicines (monotherapy) to prevent malaria parasites from developing resistance to this drug (http://www.who.int/mediacentre/news/releases/2006/pr02/en/index.html). This call comes at a time when several observations challenge the notion that artemisinin resistance does not develop. These include: (i) the development of stable resistance to artemisinin after selection pressure in an animal model [14]; (ii) the observation of *in vitro* resistance to artemether in isolates from French Guiana [15]; and (iii) the first longitudinal clinical study that demonstrated a link between reduced *in vitro* artemisinin susceptibility and loss of clinical efficacy of artesunate monotherapy [16].

The advantages of understanding the molecular basis of drug resistance are evident: monitoring drug resistance by using molecular techniques avoids the use of more cumbersome assays that depend purely on phenotypic observation. In addition, if a mutated target confers resistance, then it might be possible to design a derivative that circumvents the effects of the mutation.

Resistance to antimalarial drugs commonly arises from one of two possible mechanisms: alteration in drug disposition (usually increased drug efflux from parasites) or changes in susceptibility of drug targets. Monitoring genotypic markers of drug resistance might be applied to both disposition [17–19] and target models of resistance [20]. The target of dihydrofolate-reductase inhibitors is an excellent example where identification and structural resolution of an antimalarial target has helped develop derivatives to bypass mutations that cause resistance [21,22]. In contrast, discussion on the mechanism of action of artemisinins has been mired in detailed yet disparate hypotheses that suggest that artemisinins have multiple targets [6,23].

Thus far, the lack of evidence for artemisinin resistance has supported multiple-target models for the mechanism of action of artemisinins. Indeed, mutations that reduce artemisinin affinity would need to occur in all the targets before resistance would develop (becoming increasingly improbable as the number of targets increases). Therefore, the lack of evidence for artemisinin resistance and the multiple-target theory of artemisinin action have been self-sustaining by generating wonderfully circular arguments such as: ‘resistance to drugs that have multiple targets is less likely to develop than resistance to drugs that have a single target. Therefore, because resistance to artemisinins has not yet developed, these drugs must have multiple targets’. A critical re-evaluation of these interdependent systems will inevitably require assimilation of data from both field-based studies and basic science. Here, we will discuss some recent developments that are relevant to understand the mechanism of action of artemisinins and put them in the context of previous mechanistic arguments.

## Mechanism of action of artemisinins

Progression from chemistry experiments to validation of the mechanism of action of a drug can be a lengthy process. For example, to progress from understanding the metabolism of cholesterol to the use of statins for the treatment of hyperchloresterolaemia took ∼40 years [24,25]. Validation of drug targets (at least in the ‘pre-omics’ era) can also take years or decades because the process requires inputs from different disciplines to make a convincing case that a target is worth hitting. Reverse pharmaceutics, which identifies the target of validated drugs, might take an equally long time and not be always successful.

An endoperoxide bridge is the key pharmacophore in the semi-synthetic (and synthetic) antimalarial peroxidic compounds. 1-Carba derivatives of artemisinin are ineffective in killing parasites [26]. Many other derivatives have been studied for structure–activity relationships against parasites but as yet few systematic studies have been carried out against proposed targets. Work with enantiomerically pure 1,2,4-trioxanes that have similar potency has been regarded as evidence against a specific interaction at an enzyme active site [27]. The implication is that if protein-chiral recognition is required for this class of compounds, there should be differences between antimalarial activities of different enantiomers. This conclusion implicitly reflects the idea that C-centred radicals that are generated from endogenous ferrous iron (either free or within heme) account for the antimalarial activity of artemisinins [8]. However, this idea has not been yet adequately tested because of problems with current approaches. For example, whole-cell assays such as those performed on parasites in culture cannot account for differences that are due to differential partitioning of enantiomers into parasites or other pharmacodynamic properties that modulate drug potency. To conduct satisfactory experiments on the relevant enantiomers requires assays against proposed targets and, even then, it cannot be assured that enantiomers will elicit a different response. This depends on the nature and precision of the binding process that, with respect to the proposed PfATP6 target (see below), has not yet been precisely defined.

## The unlikelihood of heme as a target

The heme hypothesis arose in conjunction with the observation that the endoperoxide that is crucial for antimalarial activity is likely to be ‘activated’ by Fe^2+^[28], because heme is a concentrated repository for Fe^2+^. This hypothesis has been supported by other observations that include the targeting of heme by quinoline antimalarials [29] and the demonstration that artemisinin can react with heme both *in vitro* and in an animal model [30,31].

Although studies in mice have shown that labelled artemisinin is recovered in a form that is associated with heme [31], in a *Plasmodium vinckei* model the Rane test, which uses a single high dose of antimalarial to cure infection, was here used intraperitoneally at a dose of 100 mg per kilogram of artemisinin to analyse the fate of artemisinin in the presence of high parasitaemias (40–75% infected erythrocytes). In this model, recovery of heme–artemisinin was <3%. Infected red cells haemolyse and release haemozoin; therefore, it cannot be assumed that interactions between artemisinin and heme are exclusively occurring in the intra-erythrocytic context rather than after parasite release. Fluorescent artemisinin derivatives that were examined in living parasites using confocal microscopy do not localize in the parasite food vacuole that contains heme [32]. These results are consistent with classic studies that used high-resolution electron microscopy and radiolabelled artemisinin [33], where labelling was associated with intraparasitic membrane-bound structures. Others have reported that tritiated dihydroartemisinin labels the food vacuole, but close inspection of the relevant images suggests that labelling is outside food vacuoles of the parasite and not with heme [34].

Furthermore, these results are consistent with previous observations that <15% of artemisinin is in the hemozoin fraction of parasites after exposure to the radiolabelled drug [35], and that parasite stages that lack visible pigment are killed by artemisinins [2,36]. The association constants of heme and artemisinins also indicate low-affinity interaction (K_d_>10 μM) in *in vitro* studies [30]. Moreover, some artemisinin derivatives maintain antimalarial potency but cannot react with heme in conventional chemistry models of this interaction [9,10,37]. Debates over the possibility that such interactions between artemisinin and heme might occur in different chemical environments highlight the fact that, just because a particular reaction can occur, it does not mean that it actually occurs in that crucially important biological context where drug action takes place.

## Are there multiple targets for artemisinin?

The propensity for reactive intermediates formed by Fe^2+^-mediated catalysis to alkylate protein targets was explored some years ago by experiments where several parasite proteins were labelled with radiolabelled artemisinins [35]. However, the efficiency of labelling was low and it took several weeks to develop signals of labelled proteins. Partial micro-sequencing of one of these signals identified translationally controlled tumour protein (TCTP) [38], although most of the labelling was observed in an uncharacterized membrane fraction of the parasites. Other labelled proteins have yet to be identified. Interaction of tritiated dihydroartemisinin with TCTP seems to be of low affinity (K_d_>10 μM), as inferred from data presented in [38].

## Possible single targets for artemisinin

Several years ago, P-type ATPases were proposed as new targets for antimalarials. This suggestion was mainly based on the fact that selective inhibitors of P-type ATPases were already clinically used for cardiovascular and gastrointestinal disorders [39–41]. We indicated that artemisinins might inhibit the sarco–endoplasmic reticulum Ca^2+^-ATPase (SERCA) of *P. falciparum* (PfATP6) and, therefore, also SERCAs of other *Plasmodium* species because thapsigargin is a potent and selective inhibitor of SERCAs and it shares some chemical similarities with artemisinins [32]. This suggestion was substantiated by several independent experimental approaches. Artemisinins, but not other antimalarial drugs, inhibited PfATP6 activity when PfATP6 was expressed and assayed in *Xenopus* oocytes [32]. There was an excellent correlation (*R*^*2*^>0.9) between inhibitory constants of artemisinins that were assayed in the oocyte model of PfATP6 and the effectiveness of artemisinins as antimalarials that were assayed in parasite cultures. 1-Deoxyartemisinin did not inhibit PfATP6 in oocyte preparations, or kill parasites in culture unless it was used at very high concentrations (>10 μM) [32]. Artemisinin has a similar distribution compared with thapsigargin in studies on living parasites. Appropriate cross-competition studies using thapsigargin and artemisinins demonstrated that these drugs antagonise each other when they are assayed in parasite cultures [32].

Others have carried out docking simulation studies of artemisinin derivatives to models of the thapsigargin binding site in PfATP6 [42]. Several potential hydrophobic interactions between side chains of artemisinin derivatives and amino acids of PfATP6 have been identified including Leu263 (which is potentially interacting with side chain at C-12 β-position [42]). Leu263 modulated artemisinin susceptibility when examined using mutagenesis experiments of malarial SERCAs [12]. Taken together, there are independent lines of evidence that have been obtained from a range of experimental techniques to suggest that PfATP6 might be the primary target of artemisinins. However, it is suggested that genetic studies are required to support this hypothesis.

More recently, others have suggested that the electron transport chain of *P. falciparum* might be a target for artemisinins [43]. In support of this idea, when yeast was grown in non-fermentable media (making it dependent on mitochondrial respiration), sensitivity to artemisinin increased by several orders of magnitude (with IC_50_ values decreasing from 80 μM to ∼10 nM). This was accompanied by a reduction in rhodamine uptake into mitochondria. Deletion mutants *nde1Δ* and *ndi1Δ*, both of which lack the yeast NADH dehydrogenases NDE1 and NDE2, were relatively resistant to artemisinin and grew on fermentable media that contain 4 μM artemisinin [43]. Interestingly, overexpression of NDE1 and NDE2 (overexpression is inferred because they are under the regulation of an ADH1 promoter) made engineered yeast more sensitive to artemisinins than wild-type yeast. Substitution of the *P. falciparum* orthologue (PfND1I) partially restored sensitivity to artemisinins, although this effect has not yet been quantified precisely [43]. Because overexpression of some mitochondrial-transport proteins seems to increase sensitivity to artemisinins (which is contrary to what would be expected if these were targets of artemisinins because sensitivity would decrease), it has been suggested that the electron transport chain stimulates the activity of artemisinins, and that these activated artemisinins impede mitochondrial function by depolarizing mitochondrial membrane potential [43].

It is well known that artemisinin and its first-generation derivatives affect the mitochondrial inner membrane potential in neuronal-cell cultures, where interference of electron transport associated with the respiratory chain is implicated [44]. Inhibition of the inner membrane potential by artemisinins has also been described in parasites [45], where it has been shown that the respiratory chain of *Plasmodium* parasites is similar to that of mammals with regard to classic mitochondrial inhibitors of complex I–IV [45]. Artemisinin and primaquine inhibited the respiratory chain of the sexual and asexual stages of parasites [45]. The mechanism of this inhibition is unclear but it might be related to the presence of an iron group in the cytochrome center that induces the formation of radicals that seem to be unrelated to the anti-parasitic mode of action. In contrast to first generation artemisinins, artemisone has been shown to be non-neurotoxic in screens that were identical to those used to establish neurotoxicity of artemisinin and related derivatives, supporting the view that artemisone does not affect mitochondrial inner membrane potential [11].

What is the relevance of the findings in yeast models that are related to the proposed mechanism of action of artemisinins as antimalarial drugs? Parasites are incapable of aerobic glycolysis during asexual stages and there is no reported localisation of artemisinins in mitochondria. Only artemisinin itself has been tested; indeed, the pharmacophore that is crucial for antimalarial activities of artemisinins (the endoperoxide bridge) has not yet been examined by looking at the properties of 1-deoxyartemisinin. It is therefore not easy to relate findings in the yeast model to activities in parasites.

## PfATP6 as target for artemisinins: genetic evidence

In field studies of several hundred isolates that have been collected from patients with *P. falciparum* malaria since 1997, the IC_50_ of artemether of some isolates in French Guiana was greatly increased (equivalent to reduced susceptibility to this drug) when they were tested *in vitro* after short-term culture [15]. In these isolates, the IC_50_ of artemether was unrelated to the IC_50_ of other antimalarials. Sequencing of *pfATP6* identified an S769N substitution associated with this elevation in the IC_50_ value for artemether. Artemether is the only artemisinin derivative that is included in the currently registered fixed-dose artemisinin-combination therapy recommended by WHO (artemether–lumefantrine, also known as Riamet™), although other non-fixed-dose combination therapies are supposed for use. The median [interquartile (IQR)] IC_50_ value for artemether for parasites with an S769N substitution in *pfATP6* was 79.4 nM (37.2–109.3 nM), which is >20-fold higher than the upper limit of the IQR value for all parasites that have been studied in French Guiana [median (IQR) =1.7 nM (0.98–3.6 nM)]. Interestingly and worryingly, the isolates that had an increased IC_50_ value for artemether and an S769N substitution in *pfATP6* originated from areas in French Guiana that are separated by some distance by inhabited primary forest. These isolates were collected several months apart and presented distinct susceptibility profiles to a range of antimalarials. This clearly indicates that they were unrelated and, thus, that independent events were being studied. Isolates from unrelated patients examined on the same day did not exhibit an elevation in the IC_50_ value for artemether, indicating that the observed increase of IC_50_ is unlikely to be due to experimental-culture artefacts. IC_50_ assays and molecular studies were carried out independently and molecular data were generated without knowledge of the *in vitro* susceptibility profiles.

An argument that is raised against the notion that there is no artemisinin resistance is: why has resistance not arisen in areas where artemisinins have been used more heavily than in places such as French Guiana? The examples of resistance to chloroquine and sulfadoxine–pyrimethamine are helpful because, despite excessive use of these drugs and, therefore, high selection pressure in high transmission areas of sub-Saharan Africa, resistance to these drugs arose first in Southeast Asia and South America and in few other locations where population structure and transmission conditions favoured resistance [46,47,48]. Furthermore, it is not only a question of how much drug is used in a given area but also whether the drug is appropriately administered and used (e.g. given in combination with other classes of antimalarial drugs). On the Thai–Myanmar border and in Cambodia, artemisinin therapy has been given in combination with mefloquine [49]. Artemisinin resistance has not been reported in this area and no genotype–phenotype relationship has been observed for PfATP6 [50]. However, there is a risk that, if resistance to the companion drug (e.g. mefloquine) increases, this will leave artemisinins acting as monotherapies.

Antimalarial-drug pressure has been exerted on parasites in French Guiana and in surrounding areas such as Surinam and Brazil; parasites in these areas are currently resistant to several antimalarials and have mutations in many of the genes associated with multidrug resistance. These parasites have a typical South-American genetic background that differs in many drug-resistance alleles when compared with those in Southeast Asia and Africa [15].

This genetic makeup might sustain viability of parasites that carry mutations in *pfATP6* (overcoming the ‘fitness cost’ of these mutations [51]) and might explain why elevations in the IC_50_ of artemether have been observed in French Guiana. The inappropriate local use of illegally imported antimalarials that act in the genetic background of these parasites might have catalysed the emergence of drug resistance. This also might explain why adaptation to culture of these artemether-resistant isolates has proved challenging so far.

There has been no association between S769N *pfATP6* polymorphism and susceptibility to antimalarials such as mefloquine and chloroquine. Lack of associated polymorphisms to TCTP in this geographic area [15] should prompt re-evaluation of the importance of alkylation products associated with artemisinins in cultured parasites as possible targets. There is a risk that they might be ‘red herrings’ rather than informative for the mechanism of action.

The data presented by Jambou *et al.* [15] can be explained either by the altered affinity of S769N *pfATP6* for artemisinins, which directly accounts for the observed increases in the IC_50_ value, or by the fact that the altered affinity of S769N *pfATP6* for artemisinins is now so low that an alternative, lower affinity target(s) becomes biologically important. Testing the effects of this single amino acid substitution on susceptibility of *pfATP6* to artemisinins might help resolve this issue. Although the S769N substitution is located at a site of *pfATP6* that is not predicted to be associated with thapsigargin binding, it does not diminish its possible relevance to the elevation of the IC_50_ for artemisinins. In conformationally active enzymes, there are many different ways in which binding to substrates and inhibitors might be influenced by mutations that are distant to possible binding sites. In addition, the structural predictions that were made for PfATP6 are still uncertain; this enzyme has two insertions that are large compared with those in the mammalian orthologue, the crystal structure of which has been established, and that is also insensitive to artemisinins.

## Other resistance genes

In addition to mutations in *pfATP6*, other genes might modulate susceptibility to artemisinins, and these alternative mechanisms might be important. For example, increases in the copy number of *P. falciparum* multidrug resistance gene (*pfmdr1*) modulate susceptibility not only to chemically unrelated mefloquine (within a clinically relevant range of IC_50_ values) but also to artemisinins (within a small range of IC_50_ values; for example, ∼2-fold change in IC_50_ values in parasites that have been studied in Thailand) [50]. Interestingly, there were no differences in *pfmdr1* single nucleotide polymorphisms (SNPs) in isolates that were susceptible or resistant to artemether in French Guiana. The relevance of these observations to the emerging evidence of artemisinin resistance in field isolates will need to be examined further.

Animal models have also generated stable artemisinin resistance. For example, selection of genetically stable resistance to artemisinins in a model of *Plasmodium chabaudi* showed that resistance is maintained even after mosquito passage [14]. Although Afonso *et al.* [14] did not identify associated changes (either in sequence or copy number) in the SERCA orthologue of *P. chabaudi* or any other suggested marker of drug resistance in *P. falciparum* (including *pfmdr1* and *pftctp* orthologues), stable resistance to artemisinins was confirmed. These results also highlight the difficulties in translating findings from laboratory models to the study of antimalarial-drug resistance that is observed in field isolates. It might be that intrinsically less susceptible SERCAs, such as those of rodent *Plasmodium* species [12], make the emergence of alternative mechanisms for artemisinin resistance more probable and less relevant to those of *P. falciparum*. For example, a hitherto unidentified transporter that exports artemisinins might be more active in these resistant strains, and, therefore, reduce the likelihood of selection for more artemisinin-resistant variants of rodent SERCAs.

## Implications for artemisinin resistance

Although decreased susceptibility to an antimalarial drug *in vitro* does not necessarily mean therapeutic failure, it can be stated that, when clinical resistance is documented, then *in vitro* susceptibility is also decreased. The temporal scenario for the establishment of clinically relevant drug resistance might first include a decrease in *in vitro* susceptibility associated with a key mutation in the target enzyme, followed by accumulation of additional mutations, ultimately resulting in high rates of clinical failure [48]. In this way, field observations from French Guiana might represent the first element of this cascade.

These are still early days for the use of artemisinin-containing therapies; therefore, there is opportunity to assess the potential emergence of clinically significant resistance, and perhaps to try and minimise this risk. The clinical significance of decreased *in vitro* susceptibility to artemisinins needs to be explored further, although there is a possibility that, once detected, the dissemination of artemisinin-resistant parasites might be too fast to be stemmed. There have been few studies that examined the relationship between *in vitro* artemisinin susceptibility and treatment response, particularly when artemisinin-based monotherapy is employed. An interesting study from Central Africa in non-immune individuals showed that, after seven days of artesunate monotherapy [52], parasitological cure rates were 85% at day 42 of follow up (n=55 patients. In these patients, PCR-correction was used to confirm recrudescence rather than re-infection). Although the IC_50_ value of dihydroartemisinin was similar in recrudescent and cured parasites, IC_90_ values were 5-fold higher in the recrudescent group (n=5 out of 8 patients for whom data were obtained) compared with the non-recrudescent group (n=17 out of 47; *P*=0.02). This indicates that IC_50_ measurements might be a less sensitive marker of *in vitro* artemisinin resistance than IC_90_ measurements. These findings suggest that parasites with greatly increased inhibitory-constant values for artemisinins might also be more likely to relapse if artemisinin-based monotherapy is used to treat malaria [15].

This emphasizes further the need to use artemisinins in combination therapies where they act rapidly to reduce parasite numbers [1,53] rather than mediate final cure. Combining artemisinins with other antimalarials might be useful in delaying the emergence of artemisinin resistance [1], but only if parasites have not developed high-grade resistance to the companion drug [54]. Although combination therapies have provided sustained efficacy in some parts of Southeast Asia (>90% cure rates in Thailand with mefloquine–artesunate combinations [49]), there are suggestions that in other areas such as Cambodia the cure rate can fall to <80% (e.g. after artemether–lumefantrine treatment) [55]. The importance of artemisinin-combination therapy in reducing the emergence of drug-resistant parasites also needs to be explored further in areas of high endemicity of malaria. In any case, it is essential that development of new regimens is accompanied by monitoring for resistance to each drug partner using the most sensitive (often genetic) markers that are available. In this regard, recent advances in the understanding of resistance mechanisms for artemisinins and other drugs have come at a fortunate time [15,50].
